# How to measure post-error slowing: The case of pre-error speeding

**DOI:** 10.3758/s13428-021-01631-4

**Published:** 2021-07-08

**Authors:** Roland Pfister, Anna Foerster

**Affiliations:** University of Wuerzburg, Wuerzburg, Germany

**Keywords:** Post-error slowing, Pre-error speeding, Performance monitoring, Response-time analysis

## Abstract

Post-error slowing is one of the most widely employed measures to study cognitive and behavioral consequences of error commission. Several methods have been proposed to quantify the post-error slowing effect, and we discuss two main methods: The traditional method of comparing response times in correct post-error trials to response times of correct trials that follow another correct trial, and a more recent proposal of comparing response times in correct post-error trials to the corresponding correct pre-error trials. Based on thorough re-analyses of two datasets, we argue that the latter method provides an inflated estimate by also capturing the (partially) independent effect of pre-error speeding. We propose two solutions for improving the assessment of human error processing, both of which highlight the importance of distinguishing between initial pre-error speeding and later post-error slowing.

## Introduction

Research on human performance has a natural tendency to address situations in which actions go awry. Failures to enact an intended action have attracted attention from empirical researchers because they are of considerable interest for applied and basic research alike. On an applied note, understanding the conditions that give rise to errors helps to avoid adverse events in organizational settings (Reason, 1990). On a basic note, zooming in on error commission opens a window on the mechanisms that monitor performance and ensure efficient action control. The following analyses focus on this latter type of research.

Basic research on error processing has leveraged a range of behavioral and physiological markers to capture error detection and subsequent adaptations to cognition and action (Dignath et al., 2019; Falkenstein et al., 2000; Fiehler et al., 2005; Gehring et al., 2012; Steinhauser et al., 2017). Among these measures, *post-error slowing* – i.e., the observation of prolonged response times following a commission error in choice reaction tasks – comes with a particularly long history in the field. The measure of post-error slowing rose to prominence in the 1960s and 1970s (Laming, 1968, 1979; Rabbitt, 1966; Rabbitt & Rodgers, 1977) and it has continued to attract the attention of empirical researchers ever since (Crump & Logan, 2013; Danielmeier & Ullsperger, 2011; Notebaert et al., 2009; for recent observations of post-error *speeding* in certain conditions, see Damaso et al., 2020; Williams et al., 2016).

On a theoretical note, post-error slowing derives from several independent mechanisms that fall into two broad classes: adaptive and maladaptive ones (Notebaert et al., 2009; Wessel, 2018). Adaptive mechanisms include changes in information sampling to adopt a more cautious mode of responding, i.e., shifting towards slower but more accurate responses. If an error was triggered by distracting information in the environment, these mechanisms can further include focusing on task-relevant aspects of the current situation. Maladaptive mechanisms, by contrast, relate to attentional distraction and possible emotional consequences of error commission, thus yielding a negative impact on future performance. A key factor that determines the relative contribution of different adaptive and maladaptive mechanisms to post-error slowing is the timing of the current task. Maladaptive contributions are especially prominent early after error commission, whereas adaptive contributions gradually take over as time elapses (Jentzsch & Dudschig, 2009; Steinhauser et al., 2017).

But what exactly is post-error slowing in mathematical terms? The traditional method to compute this measure is

1 $$ {\varDelta}_{post\mid omnibus}=\overline{RT_{E+1}}-\overline{RT_{C+1}} $$

with $\overline{RT}$ denoting average response times, E+1 indicating correct trials that follow directly after an erroneous trial, and C+1 indicating correct trials that are preceded by another correct trial. We refer to this estimate as *Δ*_*post* ∣ *omnibus*_ because the baseline $\overline{RT_{C+1}}$ against which $\overline{RT_{E+1}}$ is evaluated comprises all correct trials following another correct trial, irrespective of when these baseline trials occur in the course of the experiment.

A relatively recent alternative proposes to adopt a different computation instead (Dutilh, van Ravenzwaaij, et al., 2012). This alternative focuses on pairs of trials immediately preceding and following an individual error:

2 $$ {\varDelta}_{post\mid E-1}=\overline{RT_{E+1}}-\overline{RT_{E-1}} $$

with E+1 again indicating correct trials following an error and E-1 indicating correct trials that immediately precede that same error.^1^ A major motivation behind this method was the claim that it might correct for fluctuations of the participant’s response time level and potentially associated differences in error frequency across the experiment (Dutilh, van Ravenzwaaij, et al., 2012; see also Hoffmann & Beste, 2015; Schroder et al., 2020). That is, in some situations, participants might commit more errors in early stages of the experiment than during later stages, e.g., because they take some time to get used to the task at hand. In other situations, they might commit more errors in the middle of the experiment, e.g., due to mind-wandering. In yet another scenario, they might commit especially many errors at the end of the experiment, e.g., due to fatigue. Similarly, the response time level of each individual participant can be expected to vary over time. If in a given situation, the likelihood of committing errors coincides with especially fast or slow responses, this correlation would bias the traditional measure of *Δ*_*post* ∣ *omnibus*_ whereas the *Δ*_*post* ∣ *E* − 1_ method would be less affected by such variation. The *Δ*_*post* ∣ *E* − 1_ method has therefore been dubbed “robust post-error slowing” upon its inception.

The aim of the *Δ*_*post* ∣ *E* − 1_ method to address overall fluctuations of the individual response time level is commendable because these variations can be sizeable at times. The pattern of this variation has further been suggested to follow similar regularities that can be observed in other complex, chaotic physical and biological systems (Gilden, 1997; Gilden et al., 1995). However, we will argue in the following that the *Δ*_*post* ∣ *E* − 1_ method is affected by a different confound that may exert an even stronger and especially a more consistent effect on the estimation of post-error slowing. This confound stems from the converse possibility of observing *pre-error speeding*, i.e., the possibility of systematically faster responses preceding an error.

## Pre-error speeding

Pre-error speeding has received considerably less attention than post-error slowing, with an exemplary Google Scholar search returning 2.750 hits for the term “post-error slowing” as compared to only 59 hits for the term "pre-error speeding" (as of December 8, 2020). In fact, the existence of pre-error speeding had been debated quite intensely in the early days of psychological research on error processing, with some researchers defending the phenomenon and others holding more skeptical positions (Laming, 1979, p. 205):

“(Rabbitt and Rodgers (1977) cite Laming (1968) to the effect that “The three responses immediately preceeding [sic] each error in continuous tasks have been found to be unusually fast”. This is incorrect. To the best of my knowledge the only evidence concerning RT on the trials before an error is that cited above from Rabbitt (1966).)”

These early discussions might foster the impression that pre-error speeding might not occur as frequently across settings and/or tasks as post-error slowing. Yet, several tasks used to study post-error slowing have been reported to feature pre-error speeding just as well if they included corresponding data in their analyses (Allain et al., 2009; Brewer & Smith, 1989; Dudschig & Jentzsch, 2009; Gehring & Fencsik, 2001; Jackson & Balota, 2012; Jentzsch & Leuthold, 2006; Murphy et al., 2016). Most studies on error processing do not report on this facet of the data, however, because assessing pre-error speeding requires a dedicated assessment of several responses surrounding an erroneous response. We will refer to these responses as *peri-error responses* for the remainder of this article. Even though assessing several peri-error responses is less compact than the common binary comparison of post-error trials and a corresponding baseline, we believe that such an approach provides useful insights.

**Fig. 1:**
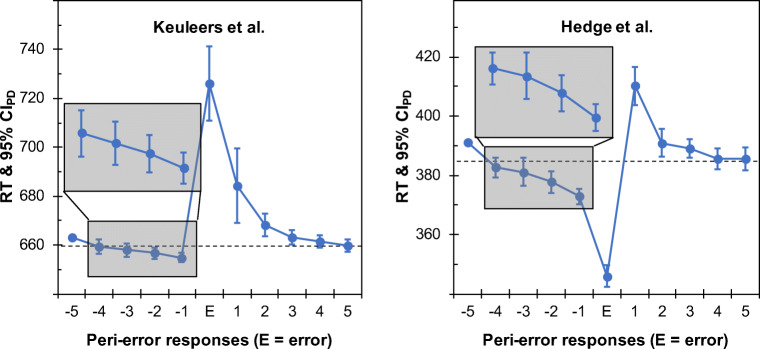
Response times (RTs) for peri-error responses in a word-nonword classification task (*left*) and a flanker task (*right*). Pre-error speeding was evident across participants in both datasets. The *dashed line* indicates the mean RT of all correct responses that were preceded by another correct response. *Error bars* show 95% confidence intervals for paired differences for each response relative to the immediately preceding response (CI_PD_; Pfister & Janczyk, 2013) so that there is a significant pairwise difference in a paired-samples *t*-test if the 95% CI_PD_ for any one mean does not include the preceding mean

To provide a snapshot of peri-error responses in different setups, we re-analyzed two different datasets by focusing on those error trials that were preceded by at least five correct trials and that were followed by at least five correct trials. This range should exceed the proposed span of three trials for pre-error speeding so that the first and last trial of this sequence should yield an unbiased estimate of the current response time level (Rabbitt, 1966). Specifically, we re-analyzed the data of Keuleers et al. (2010) who had employed a word-nonword classification task, and the data of Hedge et al. (2019) who had used a flanker task to assess cognitive control (among other tasks that we did not include in the analyses for brevity). These datasets were chosen because they include considerably high trial numbers per participant and thus provide robust estimates of corresponding peri-error response times (the former dataset had also been analyzed by Dutilh, Vandekerckhove, et al., 2012, for this reason). At the same time, the two tasks represent rather different experimental designs so that observing similar patterns in both datasets would suggest that potential findings are sufficiently generalizable. Figure 1 gives an overview of the corresponding data patterns (see the following link for a description of the underlying raw data and corresponding analysis files: https://osf.io/rd4tp/).

**Fig. 2:**
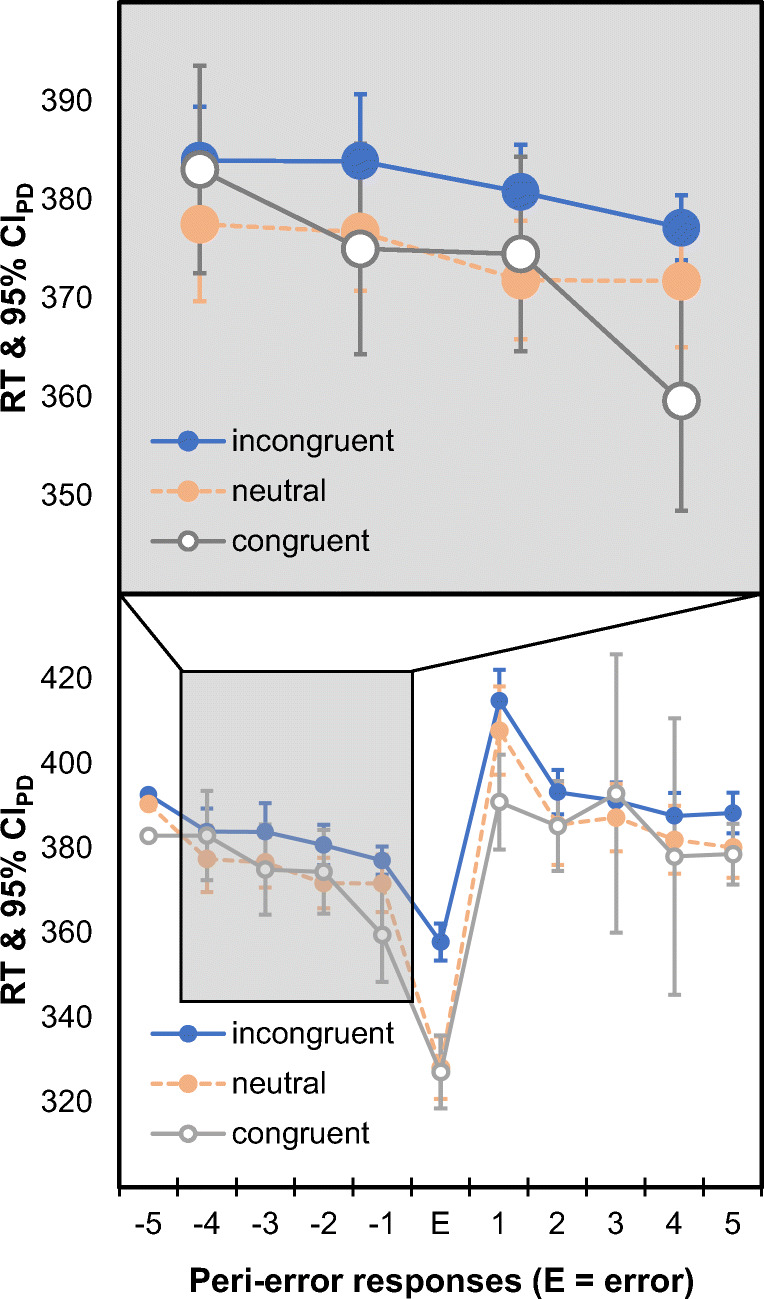
Response times (RTs) of peri-error responses in a flanker task (Hedge et al., 2019). Data are split into different trial types at the time of error commission (i.e., at time-point E on the *x*-axis). The *upper panel* provides a focus on the four trials preceding an error to make pre-error speeding more easily accessible. Target and flankers called for the same response in congruent trials, they called for different responses in incongruent trials, and the flankers did not call for any response in neutral trials. *Error bars* show 95% confidence intervals for paired differences relative to the immediately preceding response (CI_PD_; Pfister & Janczyk, 2013)

Both datasets came with pronounced post-error slowing. The two methods estimated the post-error slowing effect as *Δ*_*post* ∣ *omnibus*_ = 25.5 ms, 95% CI = [20.6; 30.3], *d*_*z*_ = 1.40, and *Δ*_*post* ∣ *E* − 1_ = 37.2 ms, 95% CI = [32.0; 42.4], *d*_*z*_ = 1.92, for the word-nonword classification data (Keuleers et al., 2010), as well as *Δ*_*post* ∣ *omnibus*_ = 24.6 ms, 95% CI = [18.9; 30.4], *d*_*z*_ = 1.39, and *Δ*_*post* ∣ *E* − 1_ = 29.3 ms, 95% CI = [23.2; 35.3], *d*_*z*_ = 1.56, for the flanker data (Hedge et al., 2019). Furthermore, comparing the response time immediately preceding the error with the response time three responses earlier yielded robust pre-error speeding in both cases: ${\varDelta}_{pre\mid E-4}=\overline{RT_{E-4}}-\overline{RT_{E-1}}$ = 4.4 ms, 95% CI = [1.6; 7.2], *d*_*z*_ = 0.50 (Keuleers et al., 2010); *Δ*_*pre* ∣ *E* − 4_ = 9.8 ms, 95% CI = [6.4; 13.2], *d*_*z*_ = 0.78 (Hedge et al., 2019). These observations document that pre-error speeding can be expected to occur quite systematically in typical laboratory tasks that are widely used to study error processing. Furthermore, pre-error speeding can vary between different conditions of an experiment as suggested by an in-depth analysis of the flanker data (Hedge et al., 2019), which can be broken down to errors that occurred in congruent trials (target and flankers indicating the same response), incongruent trials (target and flankers indicating different responses), and neutral trials (flankers did not indicate any response). Figure 2 shows that errors in congruent trials were associated with particularly pronounced pre-error speeding in this case.

At first sight, these observations might be taken to support the *Δ*_*post* ∣ *E* − 1_ method of computing post-error slowing because the trial preceding the error might be seen as the best estimate for the current response time level. This conclusion is not warranted, however, when considering that a momentary decrease in response times would be expected to be compensated by increasing response times sooner or later, irrespective of whether the decrease is followed by an error or by a correct response (see Brewer & Smith, 1989, for a related argument).

**Fig. 3:**
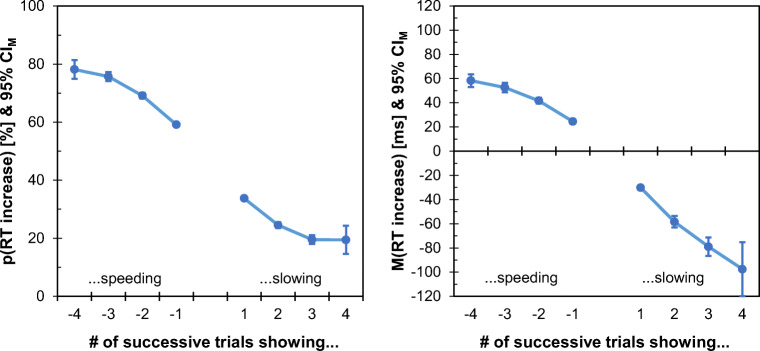
The probability of observing increasing response times (RTs) across trials and the expected mean increase as a function of the RT history in the flanker data (Hedge et al., 2019). Negative numbers on the *x*-axis reflect sequences of continued speeding across up to four consecutive trials, whereas positive numbers reflect sequences of continued slowing. All sequences included correct trials only. *Error bars* show 95% confidence intervals for the individual mean (CI_M_)

To evaluate the likelihood of observing increasing response times following a sequence of correct responses with continued speeding or slowing, we coded the flanker data (Hedge et al., 2019) according to how many preceding responses were faster or slower than their immediate predecessor. For sequences of up to four consecutive decreases or increases of response time, we further computed the probability of observing an increasing response time for the upcoming response as well as the expected magnitude of this increase in milliseconds (sequences of five or more trials of consecutive speeding or slowing were rare – only 0.2% of all coded sequences – and were therefore not included in the analyses). Figure 3 shows that already sequences of two successive speed-ups came with a probability of about 70% to observe a slower response on the next occasion.

These observations suggest that momentary speeding before an error does not reflect a proper estimate of the current response time level. Because post-error slowing aims at measuring error-induced changes on behavior – as operationalized via response times in typical choice reaction tasks – using a biased baseline will undermine the gist of any index of post-error slowing. Furthermore, even though pre-error speeding contributes to the emergence of an error in the first place (Dudschig & Jentzsch, 2009), it likely resembles an independent aspect of error commission. In fact, bivariate correlations of pre-error speeding and post-error slowing using the omnibus method amounted to *r* = – 0.01 for the word-nonword classification data and *r* = – 0.32 for the flanker data, corresponding to less than 10% shared variance in the present analyses. The trial immediately preceding an error might thus represent a relatively poor baseline for estimating the processing changes following error detection so that the measure of *Δ*_*post* ∣ *E* − 1_ can be expected to provide an overly progressive estimate of post-error slowing. It should also be noted that for both exemplary datasets, $\overline{RT_{C+1}}$ – i.e., the average response time of correct responses that follow another correct response – aligns closely with the response times observed only few trials before an error, e.g., $\overline{RT_{E-4}}$, and it also aligns closely with the response times observed only few trials following an error, e.g., $\overline{RT_{E+4}}$ (see Fig. 1). As shown in Table 1, the magnitude of *Δ*_*post* ∣ *E* − 1_ can thus be broken down into the sum of pre-error speeding and post-error slowing as evaluated by a simple omnibus method, i.e., ${\varDelta}_{pre\mid omnibus}=\overline{RT_{E-1}}-\overline{RT_{C+1}}$ and *Δ*_*post* ∣ *omnibus*_ (note that this relation holds whenever $\overline{RT_{E-1}}\le \overline{RT_C\ }\le \overline{RT_{E+1}}$ though it can be interpreted meaningfully only if $\overline{RT_{C+1}}$ aligns with the response time level at the time of error commission). Here, the effect of pre-error speeding as measured in milliseconds amounted to 18.7% and 45.9% of the following post-error slowing for the word-nonword classification data and the flanker data, respectively.

**Table 1:** Estimates for pre-error speeding and post-error slowing in milliseconds according to the omnibus method and the *Δ*_*post* ∣ *E* − 1_ method (rounded to the first decimal)

Dataset	Measure			
	*Δ*_*pre* ∣ *omnibus*_	*Δ*_*post* ∣ *omnibus*_	*Δ*_*pre* ∣ *omnibus*_+*Δ*_*post* ∣ *omnibus*_	*Δ*_*post* ∣ *E* − 1_
Keuleers et al. (2010)	4.6	24.6	29.3	29.3
Hedge et al. (2019)	11.7	25.5	37.2	37.2

The close match of $\overline{RT_{C+1}}$ and the response times about four trials before and after an error further indicates that neither dataset came with a notable bias due to correlations of error likelihood and response time level on the group level. At least for these datasets, it therefore does not seem as if the hypothesized confounds that motivated the *Δ*_*post* ∣ *E* − 1_ method actually had any systematic impact for the present data (< 1 ms for both datasets). Accordingly, a direct comparison showed pre-error speeding to affect the data more strongly than response time fluctuations as measured by the comparison of $\overline{RT_{C+1}}$ and $\left(\overline{RT_{E-4}}+\overline{RT_{E+4}}\right)/2$, and this was true for the word-nonword classification data, 95% CI = [1.4 ms; 9.1 ms], *d*_*z*_ = 0.44, as well as for the flanker data, 95% CI = [3.9 ms; 14.7 ms], *d*_*z*_ = 0.46.

## Solutions

The present analyses highlight that pre-error speeding poses a serious threat to the validity of the *Δ*_*post* ∣ *E* − 1_ method. That is, using the trial immediately preceding an error as a baseline to compute post-error slowing likely incurs an over-estimation of the effect, and this systematic confound can be observed for different experimental paradigms. This over-estimation further seems to occur quite systematically across individuals as suggested by the effect sizes observed in the present analysis. This attests a limited utility of the method: By aiming to avoid potential confounds that, if they existed, would potentially bias *Δ*_*post* ∣ *omnibus*_ to an unknown extent into an unknown direction, it accepts a confound that predictably inflates the estimate.

Researchers are thus well advised not to rely on the *Δ*_*post* ∣ *E* − 1_ method. Instead, it seems fruitful to distinguish systematically between pre-error speeding and post-error slowing in any analysis of human error processing. Construing both effects as (partially) tapping into independent psychological processes will promote more refined theorizing on performance monitoring (for a different opinion, see Schroder et al., 2020) both for healthy participants as well as for clinical populations (see, e.g., Agam et al., 2014; Polli et al., 2006; Shiels et al., 2012). Construing both effects as partially independent further allows for a more direct comparison to slowing effects in relation to unforeseen events, for which there will typically be no pre-oddball speeding (Notebaert et al., 2009; Saunders & Jentzsch, 2012). These oddball events also include observed errors (De Bruijn et al., 2012; Schuch & Tipper, 2007; Weller et al., 2018) as well as unexpected outcomes of one’s own action (Pfister et al., 2020; Steinhauser & Kiesel, 2011).

Despite this criticism of the exact computational method, we believe that the possibility of observing confounds by systematic intraindividual correlations of error likelihood and response time level has to be taken into account, however (Dutilh, van Ravenzwaaij, et al., 2012). A sequential, data-driven assessment seems to be the most promising solution in this regard.

In a first step, plotting a range of peri-error response times against $\overline{RT_{C+1}}$, i.e., the average response time of correct responses that are preceded by another correct response, allows evaluating whether a given dataset comes with substantial intra-individual correlations of error likelihood and response time level. If group effects are of interest, this assessment can focus on the group level, whereas individual assessments would be required for inter-individual difference approaches. If this first step indicates a sufficiently close match of $\overline{RT_{C+1}}$ and the overall level of peri-error response times – as estimated, e.g., by $\left(\overline{RT_{E-4}}+\overline{RT_{E+4}}\right)/2$ – then using the traditional omnibus method will return satisfactory results for pre-error speeding and post-error slowing alike.

An example for visually assessing whether or not error frequency varies systematically with response time level is shown in Fig. 1. Ideally, this method is also applied to each individual participant to address whether biases of opposing direction cancel out on the group level. A more principled way to ensure that the traditional method returns unbiased results is a comparison of a local estimate of the response time level, such as $\left(\overline{RT_{E-4}}+\overline{RT_{E+4}}\right)/2$, with the overall estimate of $\overline{RT_{C+1}}$. Because this comparison will often aim at supporting the null hypothesis of no difference, it seems useful to consider statistical approaches that explicitly allow for these conclusions, such as Bayes Factors or equivalence testing (e.g., Lakens et al., 2018; Rouder et al., 2009).

If the first step indicates a poor match of $\overline{RT_{C+1}}$ and the overall level of peri-error response times, then a less biased estimation would rely on peri-error response times that can be assumed not to be affected by either slowing or speeding relative to the error. For the present datasets, any calculation based on a larger span than *E* ± 2 replicates the results of the omnibus method as shown in Table 2.^2^ Because pre-error speeding has been proposed to affect up to three responses before an error (Rabbitt, 1966), using the fourth trial before an error might be a reasonable default ($\overline{RT_{E-4}}$). Whether it is also advisable to include a symmetrical second trial to the baseline (e.g., $\overline{RT_{E+4}}$) as we had done in the above analyses depends on the availability of sufficiently many trials with extended sequences of correct responses before *and* after an error. For the present datasets, using symmetrical baselines of *E* ± 2 to *E* ± 4 yielded a trial loss of up to 33% but comparable effect sizes in milliseconds as well as somewhat larger effect sizes in terms of Cohen’s *d*_*z*_ than using single baselines of *E* − 2 to *E* − 4 (see the Appendix for details). Should a dataset allow this stricter selection, it thus seems promising to use a symmetrical baseline because reduced variance of the baseline estimates seems to outweigh the corresponding trial loss:

3 $$ {\varDelta}_{post\mid E\pm 4}=\overline{RT_{E+1}}-\frac{\overline{RT_{E-4}}+\overline{RT_{E+4}}}{2} $$

4 $$ {\varDelta}_{pre\mid E\pm 4}=\frac{\overline{RT_{E-4}}+\overline{RT_{E+4}}}{2}-\overline{RT_{E-1}} $$

**Table 2:** Estimates for pre-error speeding and post-error slowing (in milliseconds) for different spans of peri-error response times. Please see Table 1 for comparisons to the omnibus method

Dataset	Time [t]	Measure		
		*Δ*_[*t*] ∣ *E* ± 2_	*Δ*_[*t*] ∣ *E* ± 3_	*Δ*_[*t*] ∣ *E* ± 4_
Keuleers et al. (2010)	pre	7.0	5.8	6.0
	post	20.8	22.2	22.4
Hedge et al. (2019)	pre	11.6	11.9	10.7
	post	23.2	24.0	25.2

Other variants of this approach might be more potent for specific datasets, however. It would thus be advisable to gauge for each dataset whether it is possible to use a narrower window to increase the number of trials that can be used for analysis, or whether it might even be advisable to focus on relatively distant pre-error and post-error response times when observing an extended sequence of pre-error speeding or a slow decay of post-error slowing. Using only a single baseline trial such as only $\overline{RT_{E-4}}$ or only $\overline{RT_{E+4}}$ can always be a fallback option to consider as well.

In any case, an obvious drawback of using an extended sequence of peri-error responses is that this approach requires a high number of correct trials surrounding most errors, especially as compared to the traditional method. Such data will be available in many experimental tasks, though researchers would ideally take this fact into consideration when planning the trial numbers to include in a study. This also includes the possibility that some errors will tend to occur in chunks which cannot be analyzed meaningfully with the methods at hand (e.g., Brewer & Smith, 1989; Cheyne et al., 2011; Hajcak & Simons, 2008). To give a brief example for the flanker data, 87.2% of the errors occurred as solitary events, 10.2% occurred in pairs, and 1.9% came in chunks of three. Larger chunk sizes were virtually absent (size 4: 0.5%, size 5: 0.2, size 6: 0.1%; chunks of more than 6 amounted to less than 0.1%). A dataset that comes with a high proportion of chunked errors might warrant analyses that are more tailored to the data at hand so that no off-the-shelf protocol can be provided for these cases.

While these considerations offer the chance of tailoring the analytical approach to individual datasets, they also come with the challenge of introducing researcher degrees of freedom. We therefore recommend defining a considerate plan on how to decide for a baseline (i.e., omnibus, single or symmetrical peri-error responses) based on specific criteria from the data at hand (i.e., number of cell observations, extent of pre-error speeding) before data collection.

## Conclusion

Human errors can be accompanied by systematic speeding before an error, they can be accompanied by systematic slowing in its aftermath, or they can be accompanied by both effects. Capturing pre-error speeding and post-error slowing as two distinct effects, possibly tapping into distinct psychological mechanisms, is likely to maximize theoretical insights from empirical data. Best-practice methods on how to compute these measures depend on the data at hand. If the likelihood of committing an error is statistically independent of fluctuations of an individual’s response time level across an experiment, then the traditional method of computing both measures against the overall average of correct response times following another correct response will yield satisfactory results. Alternative methods relating to peri-error response times several responses before or after the error are required if the likelihood of committing an error is correlated with fluctuations of an individual’s response time level.

## Figures and Tables

**Fig. 4:**
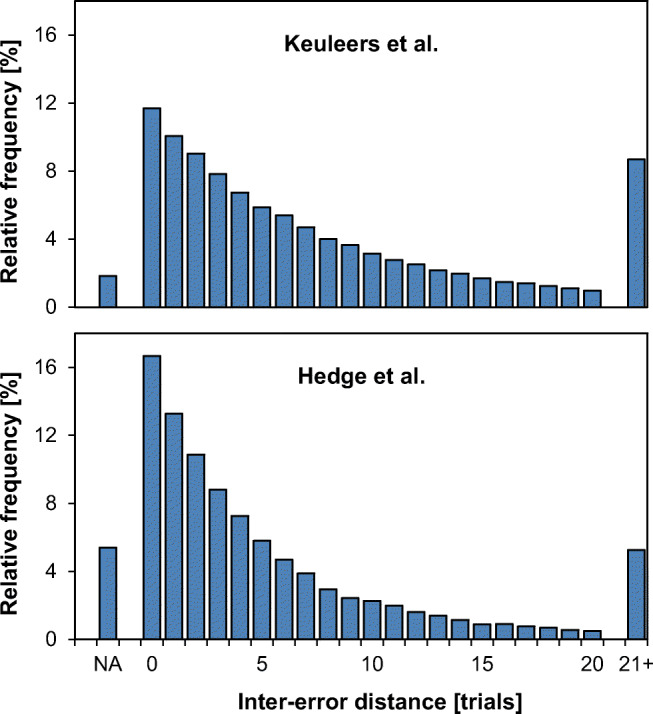
Distribution of inter-error distances for two different datasets. NA reflects trials for which no distance could be computed (here: the last trial of each block when computing distance relative to the following error), whereas 0 indicates an immediate succession of two errors

## Appendix

Any variant of computing pre-error speeding and post-error slowing based on peri-error responses will always draw on fewer trials than the traditional method. The exact number of trials depends on how quickly two error trials follow each other. Figure 4 gives an impression of the distribution of inter-error distances (measured in trials) for both datasets that were analyzed in the current study. Small inter-error distances cannot be analyzed with methods that require a certain number of correct peri-error trials. Table 3 summarizes the effect of different trial (i.e., baseline) selections on pre-error speeding and post-error slowing.

**Table 3:** Impact of different baseline selections on pre-error speeding and post-error slowing, as well as available error trials for analysis relative to all committed errors

Dataset	Time [t]	Measure					
		Symmetrical baselines			Single baselines		
		*Δ*_[*t*] ∣ *E* ± 2_	*Δ*_[*t*] ∣ *E* ± 3_	*Δ*_[*t*] ∣ *E* ± 4_	*Δ*_[*t*] ∣ *E* − 2_	*Δ*_[*t*] ∣ *E* − 3_	*Δ*_[*t*] ∣ *E* − 4_
Keuleers et al. (2010)	pre [ms]	7.04	5.79	5.98	2.55	3.79	5.10
	pre [*d*_*z*_]	1.21	0.88	0.78	0.54	0.59	0.68
	post [ms]	20.78	22.23	22.35	25.22	24.24	22.46
	post [*d*_*z*_]	1.40	1.30	1.14	1.47	1.37	1.17
	Available trials [%]	53.33	41.92	33.15	60.11	53.22	47.25
Hedge et al. (2019)	pre [ms]	11.60	11.91	10.70	6.74	10.20	12.03
	pre [*d*_*z*_]	1.38	1.08	0.91	0.72	0.80	1.00
	post [ms]	23.20	23.96	25.16	26.92	23.65	21.82
	post [*d*_*z*_]	1.58	1.41	1.48	1.64	1.34	1.31
	Available trials [%]	42.37	31.57	23.94	49.48	42.08	35.85

## Footnotes

1 The original description of the method computed the mean of the pairwise differences as compared to the difference of both paired means, as pointed out by a reviewer of an earlier version of this article (Dutilh, van Ravenzwaaij, et al., 2012). Both variants necessarily yield the same results and we use the present formula for ease of comparison to the other methods that we discuss in this article.

2 The measures shown in Table 2 were computed for different trial selections with a variable number of correct trials surrounding an error depending on the current baseline. We additionally determined that each set of peri-error trials should be preceded by a correct trial to keep the baseline free from post-error effects. The baseline of *E* ± 4 for instance is based on a trial selection with five correct trials preceding an error and four correct trials after an error. The results also replicated for a constant selection of trials with five correct responses preceding and following each error, though it seems advisable to use the shortest span possible in practice to maximize statistical power.
